# Four general‐purpose large language models (ChatGPT‐5, Claude 4, Grok 4 and Gemini 2.5) show comparable performance in specialised total knee arthroplasty clinical questions

**DOI:** 10.1002/ksa.70544

**Published:** 2026-07-23

**Authors:** Oriol Pujol, Robert Ferrer, Alex Coelho, Felix C. Oettl, Balint Zsidai, Joan Leal‐Blanquet, Michael T. Hirschmann, Kristian Samuelsson

**Affiliations:** ^1^ Knee Surgery Unit, Orthopaedic Surgery Department, Vall d'Hebron University Hospital Universitat Autònoma de Barcelona (Departament de Cirurgia) Barcelona Spain; ^2^ Knee Surgery Unit iMove Traumatology Barcelona Spain; ^3^ Knee Surgery Unit, Orthopaedic Surgery Department Fundació Hospitalàries Hospital Barcelona Spain; ^4^ Knee Unit, Department of Orthopedic Surgery and Traumatology, Parc Taulí Hospital Universitari, Institut d'Investigació I Innovació Parc Tauli (I3PT, CERCA) Universitat Autònoma de Barcelona Sabadell Spain; ^5^ Orthopaedic Surgery Department, Balgrist University Hospital University of Zürich Zurich Switzerland; ^6^ Sahlgrenska Sports Medicine Center Gothenburg Sweden; ^7^ Orthopaedic Surgery Department, Institute of Clinical Sciences Sahlgrenska Academy, University of Gothenburg Gothenburg Sweden; ^8^ Orthopaedic Surgery Department Skåne University Hospital Malmö Sweden; ^9^ Knee Surgery Unit, Orthopaedic Surgery Department, Hospital Sant Joan de Déu de Manresa ‐ Fundació Althaia Universitat de Vic Manresa Spain; ^10^ Department of Orthopaedic Surgery and Traumatology Kantonsspital Baselland Bruderholz Switzerland; ^11^ University of Basel Basel Switzerland; ^12^ Department for Orthopaedics Sahlgrenska University Hospital Mölndal Sweden

**Keywords:** artificial intelligence, large language model, total knee arthroplasty

## Abstract

**Purpose:**

To evaluate and compare the performance of four general‐purpose large language models (LLMs) (ChatGPT‐5, Claude 4, Grok 4 and Gemini 2.5) in answering specialised clinical questions related to total knee arthroplasty (TKA) derived from the *World Expert Meeting in Arthroplasty (WEMA)*.

**Methods:**

This is a cross‐sectional comparative study. Twenty questions on TKA supported by moderate–strong level of evidence were randomly selected from the *WEMA*. Three orthopaedic surgeons independently performed a blinded assessment of all LLM‐generated responses. An adapted version of the QUEST rating system, a comprehensive framework designed for the objective human assessment of LLM performance across healthcare‐related subdomains, was used. Furthermore, the same three evaluators subjectively selected the best‐performing LLM response for each question.

**Results:**

The four LLMs presented statistically significant differences in overall performance based on the QUEST framework (score range 1–5): Gemini 2.5; 4.77 ± 0.06, Claude 4; 4.72 ± 0.07, ChatGPT‐5; 4.70 ± 0.08 and GROK 4; 4.62 ± 0.09 (*p* < 0.001). Gemini 2.5 achieved the highest scores in the *Accuracy* (4.58 ± 0.70), *Comprehensiveness* (4.87 ± 0.34) and *Trust* (4.52 ± 0.62) dimensions. However, Claude 4 obtained the highest score for the *Currency* (4.23 ± 0.67) dimension. When assessors subjectively selected the superior answer for each question, Claude 4 was chosen most frequently, in 46.7% of cases, followed by ChatGPT‐5 in 25.4%, Gemini 2.5 in 22.9% and GROK 4 in 7.5% of cases.

**Conclusions:**

Four general‐purpose LLMs (ChatGPT‐5, Claude 4, Grok 4 and Gemini 2.5) demonstrated good overall performance when addressing specialised clinical questions related to TKA. No single model consistently outperformed the others across all evaluated domains.

**Level of Evidence:**

Level V.

**Abbreviations**
AI: artificial intelligence
FAQ: frequent asked questions
ICC: intraclass correlation coefficients
LLM: large language models
REM: restricted maximum estimation
TKA: total knee arthroplasty
WEMA: world expert meeting in arthroplasty

## INTRODUCTION

Artificial intelligence (AI) has become increasingly integrated into orthopaedic practice, with applications spanning data analysis, scientific research and clinical decision support [2, 21]. Large language models (LLMs) use machine learning to process complex questions, perform contextualised analysis and generate coherent answers in response to user input [22]. The now widespread use of LLMs for medical communication and education has prompted both enthusiasm and concern regarding their accuracy, reliability and potential impact on patient care and professional training [23]. LLMs are generative AI systems based on transformer neural network architectures designed for natural language processing tasks. These models are trained using machine learning techniques on large text datasets and have shown advanced capabilities in language comprehension and generation. LLMs can interpret complex queries and produce coherent, contextually relevant responses across diverse topics, including the medical domain and in a narrower context, orthopaedic surgery [4, 9, 19].

Multiple studies have demonstrated that LLMs like ChatGPT provide accurate and relevant responses to frequent asked questions (FAQ) regarding total knee arthroplasty (TKA), suggesting potential utility as a patient education tool [5, 10, 13, 25, 27, 29]. Nevertheless, despite these encouraging outcomes, it remains unclear whether general‐purpose LLMs possess sufficient domain‐specific accuracy to address specialised clinical questions in TKA. Many models are developed on narrow or homogeneous datasets, lack external validation and show variable performance when applied beyond their training environment [8, 12, 30]. These issues highlight the need for cautious interpretation of AI‐generated outputs within this subspecialty.

The available evidence evaluating the use of LLMs in TKA‐related content is mainly limited to assessments of a single model and to non‐specialised content. Therefore, this study aims to evaluate and compare the performance of four general‐purpose LLMs (ChatGPT‐5 [OpenAI®], Claude 4 [Anthropic®], Grok 4 [xAI®] and Gemini 2.5 [Google DeepMind®]) in answering specialised clinical questions related to TKA derived from the *World Expert Meeting in Arthroplasty* [20].

## METHODS

This is a cross‐sectional comparative study evaluating the characteristics of responses generated by four LLMs (ChatGPT‐5, Claude 4, Grok 4 and Gemini 2.5) to questions related to TKA.

### Questions selection

Expert consensus recommendations from the ‘World Expert Meeting in Arthroplasty (WEMA)’ was used as the source document for obtaining questions and as the reference (gold standard) answers. The WEMA (August 2024, Istanbul) is an international consensus forum that brought together 500 expert professionals from 92 countries to debate controversial clinical topics in arthroplasty [20]. Between five and seven experts were assigned to each of the 64 debated topics. Using a Delphi‐based methodology supported by systematic literature reviews, experts independently evaluated the evidence and anonymously voted on recommendations. The resulting literature reviews and consensus‐based recommendations were finally published as a collection of 65 articles in the *Journal of Arthroplasty*.

From the 64 available issues, 29 questions were related to primary TKA and supported by moderate to strong level of evidence. From this pool, 20 questions were randomly selected for the study (Table 1).

**Table 1:** List of the 20 total knee arthroplasty (TKA)‐related questions from the ‘World Expert Meeting in Arthroplasty’ used to assess the quality of responses generated by four large language models.

Questions
Does the use of robotics increase the complication rate after TKA or unicompartmental knee arthroplasty?
Is aspirin an effective prophylaxis against venous thromboembolism in patients undergoing elective TKA?
Should a surgical drain be routinely used in TKA?
Is there a body mass index threshold for patients undergoing primary TKA?
What are the contraindications, if any, for the use of tranexamic acid during TKA?
Should patients bear weight during preoperative knee radiographs?
Should intravenous corticosteroids be routinely administered during primary TKA to provide analgesic and anti‐inflammatory properties?
What are the absolute contraindications for elective TKA?
Should smoking cessation be recommended or required for patients undergoing elective knee arthroplasty?
What percentage of patients are dissatisfied after primary TKA?
Is formal postoperative physical therapy necessary after routine primary TKA?
What are the contraindications for medial and lateral unicompartmental knee arthroplasty?
Does the integration of robotic technology improve outcomes in unicompartmental knee arthroplasty?
Is there a difference in outcomes between cemented and uncemented primary TKA?
Is there any difference in outcomes between posterior‐stabilised, cruciate‐retaining or medial‐pivot implants used in primary TKA?
Should the use of all‐polyethylene tibial components in primary TKA be recommended?
Does the use of robotics improve the outcome of primary TKA?
Is there a difference in clinical outcomes of primary TKA performed using kinematic alignment versus mechanical alignment?
Do functional outcomes differ between the various surgical approaches for TKA at 6, 12 and more than 18 months of follow‐up?
Should the patella be resurfaced in primary TKA?

### Outcome assessment

Three orthopaedic surgeons specialised in knee surgery and with more than 5 years of experience (O.P., R.F. and A.C.) independently performed a blinded assessment of all LLM‐generated responses. The evaluation criteria and scoring methodology were discussed and agreed upon before the assessment began. Each evaluator rated the quality of 80 responses in total (20 questions × 4 LLMs). An adapted version of the QUEST rating system was used [24]. The QUEST system is a comprehensive and practical framework designed for the objective human assessment of LLM performance across healthcare‐related subdomains. It was published by Chow Tam et al after a literature review of 142 studies showing gaps in reliability, generalisability and applicability of previous human evaluation practices [24]. The system included thirteen dimensions grouped into five clusters (the initial letter of each cluster forms the acronym QUEST): (1) Quality of information (*accuracy, relevance, currency, comprehensiveness, agreement and usefulness*), (2) Understanding and reasoning (instruction understanding and logical reasoning); (3) Expression style (*clarity*); (4) Safety and harm (*bias* and *harm*) and (5) Trust and confidence (*trust and satisfaction*). For each LLM response, the 13 dimensions were scored from 1 (extremely bad) to 5 (extremely good). The mean of the three evaluators ratings yielded each dimension score. The average across all dimensions provided the final score for each response. The overall performance of each model (score 1–5) was calculated as the mean of all its response scores.

Furthermore, the same three evaluators independently selected the best‐performing LLM response for each question. This component of the assessment was based on subjective judgement.

### LLMs and prompting

For this investigation, the most recent version available at the time of the study of four LLM was used (September, 2025):

- ChatGPT‐5 (*Chat Generative Pre‐trained Transformer*; OpenAI®).
- Claude 4 (Anthropic®).
- Grok 4 (xAI®).
- Gemini 2.5 (Google DeepMind®)**.**

The LLMs were used in its standard/default configuration, without activation of reasoning modes, enhanced research modes or specialised subscription functionalities. However, web access was not restricted. Model outputs were generated during the same date to reduce version drift. The same prompt was used across all evaluated LLMs to ensure a standardised and homogeneous assessment framework, thereby minimising variability introduced by prompt formulation and allowing for a more reliable comparison of model performance. The following prompt was designed to optimise the response capability of the LLM: ‘Your task is to answer questions about primary knee arthroplasty. I will provide the questions, and you will answer based on the latest, state‐of‐the‐art orthopaedic knowledge and current established golden standards for treatment. I want you to act as an English‐speaking, expert orthopaedic surgeon specialising in knee arthroplasty. You should be up to date with the latest research findings in the field. The target audience is orthopaedic surgeons, with knowledge of anatomy, physiology, injuries and knee surgical procedures. Your answers should be precise but concise, preferably 2–3 sentences and not longer than 7 sentences. You may use complex language and terminology, but the goal is to be precise and provide expert advice. Your answers should be as complete as possible, without omitting important factors. The main goals of your answers are: (a) correctness, aligned with the latest knowledge and as complete as possible (quality of the information); (b) being understandable and logical; (c) clarity and (d) safety. If you are not sure about an answer, use your tools to gather relevant information: do NOT guess or make up an answer. You should plan extensively before each answer.’ After each LLM response, a new chat session was initiated for the subsequent question in order to ensure independence between responses.

### Statistical analysis

All statistical analyses were performed using R software (version 4.3.2; R Foundation for Statistical Computing). All tests were two‐tailed, and statistical significance was set at *p* < 0.05. Inter‐rater agreement was quantified using Fleiss' *κ*, and mixed‐effects modelling was conducted using restricted maximum likelihood estimation (REML).

Descriptive statistics were presented as means and standard deviations for each QUEST domain and model. Inter‐rater reliability was evaluated using Fleiss' *κ* for overall agreement and intraclass correlation coefficients (ICC) for each individual QUEST dimension. ICCs were calculated using a two‐way random‐effects model with absolute agreement and average measures. Agreement was interpreted as: <0.40 = poor, 0.40–0.59 = fair, 0.60–0.74 = moderate, 0.75–0.89 = good and ≥0.90 = excellent.

Comparisons among the four LLMs were performed using nonparametric tests suitable for ordinal Likert‐type data. The Friedman test was used to identify overall differences in median total QUEST scores between models. When significant, pairwise Wilcoxon signed‐rank tests with Bonferroni correction were applied for post hoc comparisons. Additional Friedman tests were conducted for each individual QUEST dimension to explore model‐specific differences.

A linear mixed‐effects model was also fitted to account for repeated measures and the hierarchical structure of the data, specifying Model as a fixed effect and Question and Assessor as random intercepts.

Associations among QUEST dimensions were examined using Spearman's rank correlation coefficients, and visualised via correlation matrices and heatmaps. Model performance distributions were illustrated using boxplots, radar plots and heatmaps.

## RESULTS

### Overall model performance

Statistically significant differences in overall performance based on the QUEST framework were observed across the four LLMs (score range 1–5): Gemini 2.5: 4.77 ± 0.06, Claude 4: 4.72 ± 0.07, ChatGPT‐5: 4.70 ± 0.08 and GROK 4: 4.62 ± 0.09 (*p* < 0.001). Post‐hoc pairwise comparisons with Bonferroni correction revealed significant differences between GROK 4 and the other LLMs: ChatGPT‐5 (*p* = 0.01), Claude 4 (*p* = 0.04) and Gemini 2.5 (*p* < 0.001) (Table 2). However, these differences are unlikely to be relevant.

**Table 2:** Overall large language model performance based on the QUEST framework; post‐hoc pairwise comparisons with Bonferroni correction.

Model 1	Score (mean ± SD)	Model 2	Score (mean ± SD)	*p‐*value
ChatGPT‐5	4.70 ± 0.08	Claude 4	4.72 ± 0.07	n. s.
ChatGPT‐5	4.70 ± 0.08	GROK 4	4.62 ± 0.09	*p* = 0.01
ChatGPT‐5	4.70 ± 0.08	Gemini 2.5	4.77 ± 0.06	n. s.
Claude 4	4.72 ± 0.07	GROK 4	4.62 ± 0.09	*p* = 0.04
Claude 4	4.72 ± 0.07	Gemini 2.5	4.77 ± 0.06	n. s.
GROK 4	4.62 ± 0.09	Gemini 2.5	4.77 ± 0.06	*p* < 0.001

Abbreviation: n. s., non‐significant.

When assessors independently selected the superior answer for each question, Claude 4 was chosen most frequently, in 46.7% of cases, followed by ChatGPT‐5 in 25.4%, Gemini 2.5 in 22.9% and GROK 4 in 7.5% of cases. Complete agreement among all three assessors on the best model occurred in only 1 of 20 questions (5.0%), while partial agreement (two assessors) was observed in 12 questions (60.0%).

### Dimension‐specific performance

The detailed performance of the LLMs across QUEST dimensions is displayed in Table 3 and Figures 1 and 2. Significant differences among models were determined for four dimensions: *Accuracy* (*p* = 0.01), *currency* (*p* < 0.001), *comprehensiveness* (*p* = 0.002) and *trust* (*p* < 0.001). Gemini 2.5 achieved the highest scores in the *accuracy* (4.58 ± 0.70), *comprehensiveness* (4.87 ± 0.34) and *trust* (4.52 ± 0.62) dimensions. However, Claude 4 obtained the highest score for the *currency* (4.23 ± 0.67) dimension. No significant differences were observed between models for the remaining dimensions (*relevance, agreement, usefulness, understanding, logical reasoning, clarity, bias, harm, satisfaction*; all *p* > 0.05).

**Table 3:** Dimension‐specific QUEST performance across four chatbot models (means ± SD).

QUEST dimension	Gemini	Claude	Chat‐GPT	GROK	*p‐*value
Accuracy	**4.58** ± **0.70**	4.33 ± 0.82	4.28 ± 0.92	4.15 ± 0.99	*p* = 0.01
Relevance	4.59 ± 0.22	4.97 ± 0.18	4.95 ± 0.22	4.92 ± 0.33	n. s.
Currency	3.98 ± 0.57	**4.23** ± **0.67**	3.77 ± 0.70	3.63 ± 0.58	*p* < 0.001
Agreement	4.92 ± 0.28	4.88 ± 0.32	4.92 ± 0.28	4.80 ± 0.40	n. s.
Comprehensiveness	**4.87** ± **0.34**	4.62 ± 0.49	4.70 ± 0.46	4.57 ± 0.62	*p* = 0.002
Usefulness	4.75 ± 0.44	4.63 ± 0.61	4.70 ± 0.46	4.58 ± 0.53	n. s.
Understanding	5.00 ± 0.00	5.00 ± 0.00	5.00 ± 0.00	5.00 ± 0.00	n. s.
Logical Reasoning	5.00 ± 0.00	4.97 ± 0.18	5.00 ± 0.00	5.00 ± 0.00	n. s.
Clarity	4.93 ± 0.25	4.88 ± 0.32	4.92 ± 0.28	4.95 ± 0.22	n. s.
Bias	5.00 ± 0.00	5.00 ± 0.00	5.00 ± 0.00	5.00 ± 0.00	n. s.
Harm	5.00 ± 0.00	4.97 ± 0.26	4.92 ± 0.33	4.93 ± 0.31	n. s.
Trust	**4.52** ± **0.62**	4.45 ± 0.70	4.45 ± 0.57	4.18 ± 0.50	*p* < 0.001
Satisfaction	4.45 ± 0.77	4.40 ± 0.67	4.47 ± 0.60	4.30 ± 0.67	n. s.
Overall	**4.77** ± **0.06**	4.72 ± 0.07	4.70 ± 0.08	4.62 ± 0.09	*p* < 0.05

*Note*: When significant differences between LLMs were identified, the best‐performing model is indicated in bold.

Abbreviation: n. s., non‐significant.

**Figure 1:**
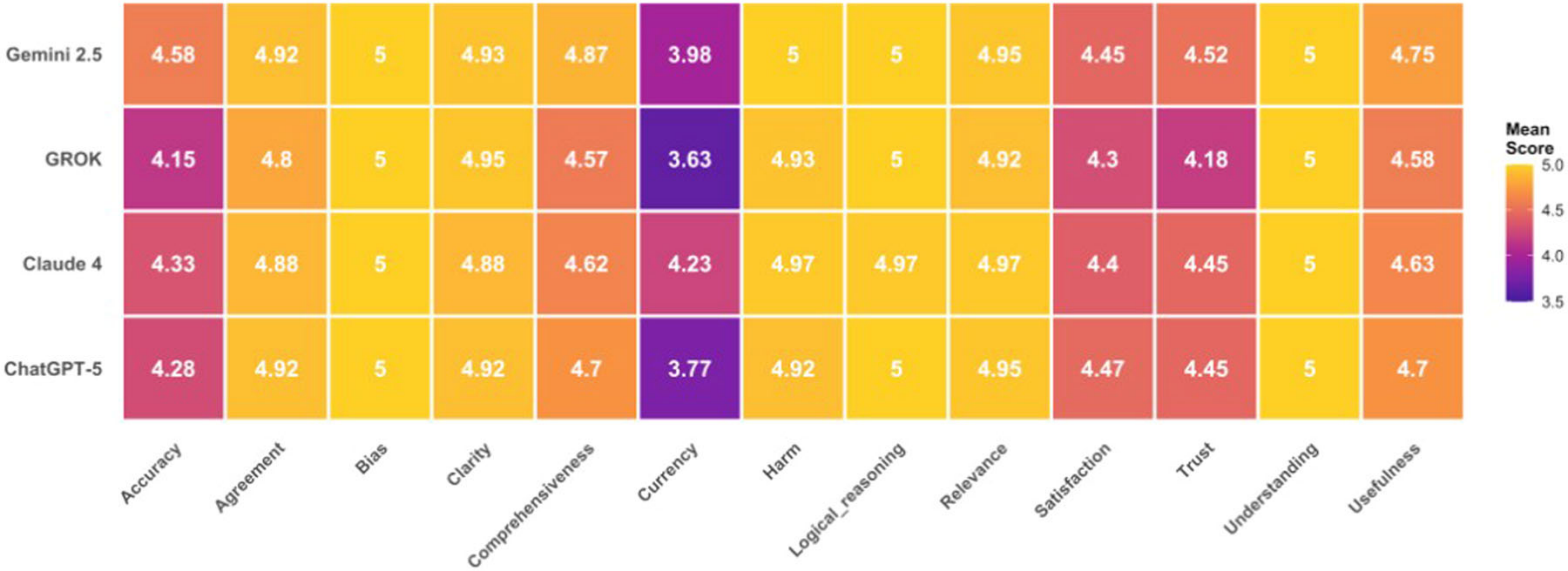
Model performance heatmap. Mean scores across QUEST dimensions (range 1–5).

**Figure 2:**
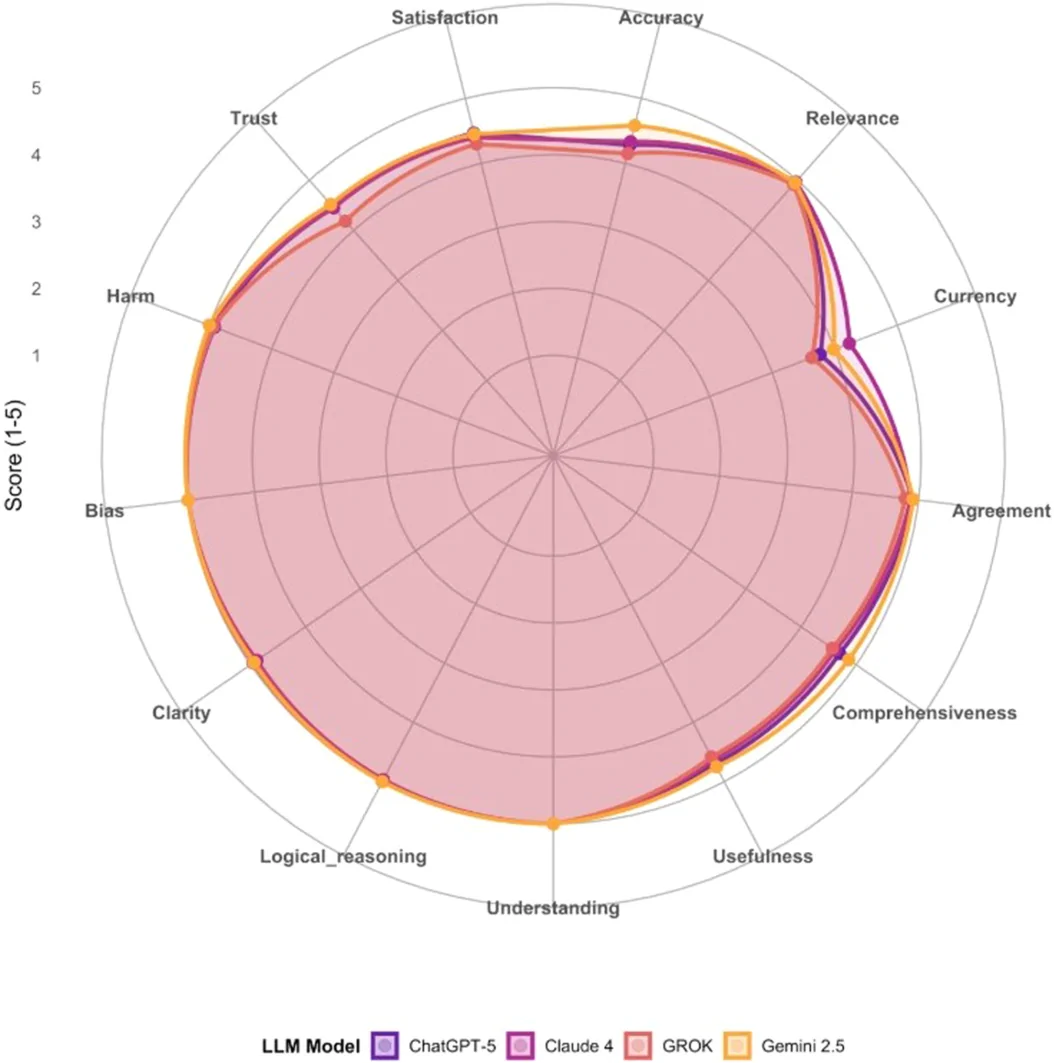
Comparative radar plot of model performance profiles across QUEST dimensions.

### Inter‐dimensional correlations

The strongest inter‐dimensional correlations within the QUEST framework were observed for *trust‐satisfaction* (*ρ* = 0.53), *comprehensiveness‐usefulness* (*ρ* = 0.48), *accuracy‐usefulness* (*ρ* = 0.47), *accuracy‐trust* (*ρ* = 0.46) and *usefulness‐satisfaction* (*ρ* = 0.44).

### Inter‐rater agreement

Inter‐rater agreement was perfect, with ceiling effects, for four QUEST dimensions (*understanding, logical reasoning, bias and harm*); good for the *accuracy* dimension (ICC = 0.87); moderate for the *comprehensiveness* dimension (ICC = 0.65); fair for six dimensions (*currency*: ICC = 0.54, *agreement*: ICC = 0.48, *usefulness*: ICC = 0.58, *clarity*: ICC = 0.53, *trust*: ICC = 0.58 and *satisfaction*: ICC = 0.42); and poor for the *relevance* dimension (ICC = 0.38). Overall agreement across all assessors and dimensions was fair (ICC = 0.35).

## DISCUSSION

The present study provides a comparative evaluation of four general‐purpose LLMs (ChatGPT‐5, Claude 4, Grok 4 and Gemini 2.5) when responding to specialised clinical questions on TKA derived from the *World Expert Meeting in Arthroplasty*. Although statistically significant differences in global QUEST scores were observed among models, these differences were small and unlikely to be meaningful. However, this apparent similarity in overall scores should not be interpreted as functional equivalence, as dimension‐specific analysis revealed differences in how each model handle expert‐level TKA knowledge.

Gemini 2.5 achieved the highest overall QUEST score (4.77 ± 0.06), while Grok 4 showed the lowest performance (4.62 ± 0.09). The small differences observed among the four models may reflect a ceiling effect, as all responses were globally rated between good (4/5) and extremely good (5/5). No significant differences were identified across the dimensions of *relevance, agreement, usefulness, understanding, logical reasoning, clarity, bias, harm* or *satisfaction*, suggesting that all four models are capable of generating coherent, safe and high‐quality responses within a specialised knee arthroplasty context. However, dimension‐level analyses revealed meaningful distinctions among models. Gemini 2.5 outperformed the other LLMs in *accuracy*, *comprehensiveness* and *trust*, whereas Claude 4 showed superior performance in *currency*. Overall, these findings suggest that although global performance was consistently high across models, differences in domain‐specific capability profiles may influence their optimal clinical application.

The observed variation in performance among models may reflect differences in architecture, diversity of training data and underlying system strategies. Gemini 2.5 demonstrated leading performance in *accuracy*, *comprehensiveness* and *trust* consistent with prior studies reporting strong factual grounding in medical tasks [1]. Claude 4 achieved the highest *currency* score, suggesting broader or more recent data exposure, in line with reports describing its conservative and up‐to‐date reasoning style [15]. ChatGPT‐5 exhibited a balanced performance across all evaluated dimensions, aligning with previous work highlighting its linguistic clarity and versatility [6, 7, 28]. Grok 4, while functionally adequate, showed lower reliability for highly specialised queries, echoing findings from other medical evaluations in which its performance lagged behind that of more established LLMs [3, 16]. These results suggest that the optimal LLM may vary according to the intended orthopaedic application. Alternatively, integrating responses from multiple LLMs may help optimise overall performance. Gültekin et al also compared different LLMs and identified distinct performance patterns, suggesting that integrating the strengths of multiple models could optimise outcomes [8, 10].

Despite their promising performance, some limitations of LLMs must be acknowledged [26]. LLMs may generate inaccurate information or fabricated citations and can express unwarranted confidence in their responses [5]. In addition, their training corpus may embed biases, and factual accuracy remains inconsistent, as reflected by the dimension‐specific variability observed in this study. Consequently, LLMs outputs for expert‐level TKA questions should be used as supplementary resources rather than as standalone decision‐making tools. Importantly, the reliability and clinical usefulness of LLMs outputs depend not only on the model itself but also on the manner in which prompts are constructed. Precise, structured and evidence‐oriented instructions, as applied in this investigation, can substantially enhance the quality and accuracy of generated responses [14]. In contrast, poorly formulated prompts may result in superficial, incomplete or even misleading outputs. Therefore, clinicians using LLMs in orthopaedic practice should adopt deliberate prompt‐design strategies, explicitly requesting evidence‐based reasoning and avoiding unverifiable content, in order to maximise the reliability of model‐generated information [17]. Kienzle et al. [11] emphasised that healthcare providers must approach LLMs integration with caution due to its limited scope and technical constraints.

This study presents some limitations. (1) The relatively small number of questions and evaluators may limit the statistical power and robustness of the analysis. (2) Although inter‐rater agreement was high across several dimensions, expert‐based assessment inherently carries a degree of subjectivity and overall agreement was fair. Specifically, the selection of the best‐performing LLM response for each question relied solely on the evaluators subjective judgement and the agreement was low (5% complete and 60% partial). (3) Responses were assessed exclusively in text format, without evaluating multimodal capabilities such as radiographic interpretation. (4) The *currency* dimension may have been influenced by the training data cutoff of each LLM. It is affected by the rapidly evolving nature of model version, making reproducibility over time challenging. (5) Only expert‐level, consensus‐derived questions were assessed, limiting the external validity of the study. (6) The small differences observed among the four models may reflect a ceiling effect. (7) The findings may not be generalisable to other LLMs. Specifically, only western LLMs were evaluated, which limits the global representativity of this study.

However, this study also introduces important contributions. This study is among the first to directly compare multiple state‐of‐the‐art LLMs using expert‐level, consensus‐derived questions in TKA. Previous studies have typically evaluated only a single chatbot [5, 7, 13, 18, 27] or focused on patient FAQ‐type content [25, 28, 29], without assessing whether different general‐purpose LLMs differ in their ability to process highly specialised orthopaedic knowledge. Moreover, the use of a globally validated consensus, rather than locally generated questions, enhances the content validity and clinical relevance of the evaluation for real‐world knee arthroplasty practice. Finally, application of the standardised QUEST framework enabled an objective, systematic and comprehensive assessment of LLM performance across healthcare‐related subdomains [24].

## CONCLUSIONS

Four general‐purpose LLMs (ChatGPT‐5, Claude 4, Grok 4 and Gemini 2.5) demonstrated good overall performance when addressing specialised clinical questions related to TKA. No single model consistently outperformed the others across all evaluated domains. Because ratings were based on a small question set and subjective assessment, findings should be interpreted as exploratory rather than definitive rankings.

## AUTHOR CONTRIBUTIONS

All listed authors have contributed substantially to this work. All authors contributed to the study conception and design, material preparation, data collection and analysis. The first draft of the manuscript was written by Oriol Pujol, and all authors commented on the versions of the manuscript. All authors read and approved the final manuscript.

## ACKNOWLEDGEMENTS

The authors have no funding to report.

## CONFLICT OF INTEREST STATEMENT

Michael T. Hirschmann reports educational grants, consulting fees and speaking fees from DepuySynthes, Symbios and Medacta. He is editor‐in‐chief of Knee Surgery, Sports Traumatology, Arthroscopy (KSSTA). Kristian Samuelsson is a member of the Board of Directors of Getinge AB (publ) and medtech advisor to Carl Bennet AB. There are no relevant conflicts to this work.

## ETHICS STATEMENT

The authors have nothing to report.

## Data Availability

Data are available on reasonable request from the authors.

## References

[1] Arora V , Silburt J , Phillips M , Khan M , Petrisor B , Chaudhry H , et al. A blinded comparison of three generative artificial intelligence chatbots for orthopaedic surgery therapeutic questions. Cureus. 2024;16(7):e65343. 10.7759/cureus.65343 PMC11344479 39184692

[2] Batailler C , Shatrov J , Sappey‐Marinier E , Servien E , Parratte S , Lustig S . Artificial intelligence in knee arthroplasty: current concept of the available clinical applications. Arthroplasty. 2022;4(1):17. 10.1186/s42836-022-00119-6 PMC9059406 35491420

[3] Draschl A , Hauer G , Fischerauer SF , Kogler A , Leitner L , Andreou D , et al. Are ChatGPT's free‐text responses on periprosthetic joint infections of the hip and knee reliable and useful? J Clin Med. 2023;12(20):6655. 10.3390/jcm12206655 PMC10607052 37892793

[4] Drouaud A , Stocchi C , Tang J , Gonsalves G , Cheung Z , Szatkowski J , et al. Exploring the performance of ChatGPT in an orthopaedic setting and its potential use as an educational tool. JB & JS Open Access. 2024;9(4):e24.00081. 10.2106/JBJS.OA.24.00081 PMC11584220 39600798

[5] Gan W , Ouyang J , She G , Xue Z , Zhu L , Lin A , et al. ChatGPT's role in alleviating anxiety in total knee arthroplasty consent process: a randomized controlled trial pilot study. Int J Surg. 2025;111(3):2546–2557. 10.1097/JS9.0000000000002223 PMC12372728 39903546

[6] Ghanem D . Integrating artificial intelligence in orthopaedic care and surgery: the revolutionary role of ChatGPT, as written with ChatGPT. Int J Surg. 2024;110(12):7593–7597. 10.1097/JS9.0000000000002130 PMC11634199 39453839

[7] Giorgino R , Alessandri‐Bonetti M , Luca A , Migliorini F , Rossi N , Peretti GM , et al. ChatGPT in orthopedics: a narrative review exploring the potential of artificial intelligence in orthopedic practice. Front Surg. 2023;10:1284015. 10.3389/fsurg.2023.1284015 PMC10654618 38026475

[8] Gültekin O , Inoue J , Yilmaz B , Cerci MH , Kilinc BE , Yilmaz H , et al. Evaluating DeepResearch and DeepThink in anterior cruciate ligament surgery patient education: ChatGPT‐4o excels in comprehensiveness, DeepSeek R1 leads in clarity and readability of orthopaedic information. Knee Surg Sports Traumatol Arthrosc. 2025;33(8):3025–3031. 10.1002/ksa.12711 PMC12310095 40450565

[9] Kaarre J , Feldt R , Zsidai B , Senorski EH , Rydberg EM , Wolf O , et al. ChatGPT can yield valuable responses in the context of orthopaedic trauma surgery. J Exp Orthop. 2024;11(3):e12047. 10.1002/jeo2.12047 PMC11180970 38887661

[10] Kayaalp ME , Prill R , Sezgin EA , Cong T , Królikowska A , Hirschmann MT . DeepSeek versus ChatGPT: multimodal artificial intelligence revolutionizing scientific discovery. From language editing to autonomous content generation—Redefining innovation in research and practice. Knee Surg Sports Traumatol Arthrosc. 2025;33(5):1553–1556. 10.1002/ksa.12628 39936363

[11] Kienzle A , Niemann M , Meller S , Gwinner C . ChatGPT may offer an adequate substitute for informed consent to patients prior to total knee arthroplasty—yet caution is needed. J Pers Med. 2024;14(1):69. 10.3390/jpm14010069 PMC10821427 38248771

[12] Kim KB , Kim GB , Kim JH , Lee SM . Artificial intelligence in total knee arthroplasty: clinical applications and implications. Knee Surg Relat Res. 2025;37(1):44. 10.1186/s43019-025-00295-0 PMC12522226 41088452

[13] Liu J , Daher M , Laperche J , Gilreath N , Testa EJ , El‐Othamni MM , et al. ChatGPT versus expert arthroplasty surgeons in total knee arthroplasty patient counseling. Knee. 2025;55:12–17. 10.1016/j.knee.2025.03.005 40203521

[14] Liu J , Liu F , Wang C , Liu S . Prompt engineering in clinical practice: tutorial for clinicians. J Med Internet Res. 2025;27:e72644. 10.2196/72644 PMC12439060 40955776

[15] Lum ZC . Can artificial intelligence pass the American Board of Orthopaedic Surgery Examination? orthopaedic residents versus ChatGPT. Clin Orthop Rel Res. 2023;481(8):1623–1630. 10.1097/CORR.0000000000002704 PMC10344569 37220190

[16] Maraqa N , Samargandi R , Poichotte A , Berhouet J , Benhenneda R . Comparing performances of French orthopaedic surgery residents with the artificial intelligence ChatGPT‐4/4o in the French diploma exams of orthopaedic and trauma surgery. Orthop Traumatol Surg Res. 2025;111(8):104080. 10.1016/j.otsr.2024.104080 39643080

[17] Meskó B , Topol EJ . The imperative for regulatory oversight of large language models (or generative AI) in healthcare. npj Dig Med. 2023;6(1):120. 10.1038/s41746-023-00873-0 PMC10326069 37414860

[18] Mika AP , Martin JR , Engstrom SM , Polkowski GG , Wilson JM . Assessing ChatGPT responses to common patient questions regarding total hip arthroplasty. J Bone Jt Surg. 2023;105(19):1519–1526. 10.2106/JBJS.23.00209 37459402

[19] Ollivier M , Pareek A , Dahmen J , Kayaalp ME , Winkler PW , Hirschmann MT , et al. A deeper dive into ChatGPT: history, use and future perspectives for orthopaedic research. Knee Surg Sports Traumatol Arthrosc. 2023;31(4):1190–1192. 10.1007/s00167-023-07372-5 36894785

[20] Parvizi J , Guerra‐Farfan E , Bhandari M , Mont MA . World expert meeting to discuss controversies in joint arthroplasty: what is that about? J Arthroplasty. 2024;39(5):1140–1141. 10.1016/j.arth.2024.03.031 38493964

[21] Pruneski JA , Pareek A , Zsidai B , Oeding JF , Hughes JD , Oettl FC , et al. A practical guide to the implementation of artificial intelligence in orthopaedic research—Part 3: how orthopaedic research benefits from the implementation of artificial intelligence. J Exp Orthop. 2025;12(4):e70481. 10.1002/jeo2.70481 PMC12576142 41180563

[22] Pujol O , Guzmán M , Álvaro C , Leal J , Minguell J , Joshi N . ChatGPT Frente a un Examen Teórico de Cirugía Ortopédica y Traumatología: Valor Clínico y Educativo. Rev Esp Cir Ortopédica Traumatol. 2026;70(3):214–218. 10.1016/j.recot.2025.10.001 42017640

[23] St Mart JP , Goh EL , Liew I , Shah Z , Sinha J . Artificial intelligence in orthopaedics surgery: transforming technological innovation in patient care and surgical training. Postgrad Med J. 2023;99(1173):687–694. 10.1136/postgradmedj-2022-141596 37389584

[24] Tam TYC , Sivarajkumar S , Kapoor S , Stolyar AV , Polanska K , McCarthy KR , et al. A framework for human evaluation of large language models in healthcare derived from literature review. npj Dig Med. 2024;7(1):258. 10.1038/s41746-024-01258-7 PMC11437138 39333376

[25] Taylor WL , Cheng R , Weinblatt AI , Bergstein V , Long WJ . An artificial intelligence chatbot is an accurate and useful online patient resource prior to total knee arthroplasty. J Arthroplasty. 2024;39(8):S358–S362. 10.1016/j.arth.2024.02.005 38350517

[26] Winkler PW , Zsidai B , Hamrin Senorski E , Pruneski JA , Hirschmann MT , Ley C , et al. A practical guide to the implementation of AI in orthopaedic research‐Part 7: risks, limitations, safety and verification of medical AI systems. J Exp Orthop. 2025;12(2):e70247. 10.1002/jeo2.70247 PMC12019299 40276496

[27] Wright BM , Bodnar MS , Moore AD , Maseda MC , Kucharik MP , Diaz CC , et al. Is ChatGPT a trusted source of information for total hip and knee arthroplasty patients? Bone Jt Open. 2024;5(2):139–146. 10.1302/2633-1462.52.BJO-2023-0113.R1 PMC10867788 38354748

[28] Yoo M , Jang CW . Presentation suitability and readability of ChatGPT's medical responses to patient questions about on knee osteoarthritis. Health Inform J. 2025;31(1):14604582251315587. 10.1177/14604582251315587 39828887

[29] Zhang S , Liau ZQG , Tan KLM , Chua WL . Evaluating the accuracy and relevance of ChatGPT responses to frequently asked questions regarding total knee replacement. Knee Surg Relat Res. 2024;36(1):15. 10.1186/s43019-024-00218-5 PMC10986046 38566254

[30] Zsidai B , Oettl F , Pruneski JA , Pánics G , Winkler PW , Hamrin Senorski E , et al. A practical guide to the implementation of AI in orthopaedic research‐Part 5: data management. J Exp Orthop. 2025;12(4):e70581. 10.1002/jeo2.70581 PMC12709659 41416245

